# Plasma contact factors as novel biomarkers for diagnosing Alzheimer’s disease

**DOI:** 10.1186/s40364-020-00258-5

**Published:** 2021-01-09

**Authors:** Jung Eun Park, Do Sung Lim, Yeong Hee Cho, Kyu Yeong Choi, Jang Jae Lee, Byeong C. Kim, Kun Ho Lee, Jung Sup Lee

**Affiliations:** 1 Department of Biomedical Science, College of Natural Sciences, Chosun University, 309 Pilmun-Daero, Gwangju, 61452 Republic of Korea; 2 Department of Integrative Biological Sciences & BK21-Four Educational Research Group for Age-associated Disorder Control Technology, Chosun University, Gwangju, Republic of Korea; 3 Gwangju Alzheimer’s disease and related Dementias Cohort Center, Chosun University, Gwangju, Republic of Korea; 4 Department of Neurology, Chonnam National University Medical School, Gwangju, Republic of Korea

**Keywords:** Alzheimer’s disease, Biomarkers, Contact factor, FXIIa, Plasma

## Abstract

**Background:**

Alzheimer’s disease (AD) is the most common cause of dementia and most of AD patients suffer from vascular abnormalities and neuroinflammation. There is an urgent need to develop novel blood biomarkers capable of diagnosing Alzheimer’s disease (AD) at very early stage. This study was performed to find out new accurate plasma diagnostic biomarkers for AD by investigating a direct relationship between plasma contact system and AD.

**Methods:**

A total 101 of human CSF and plasma samples from normal and AD patients were analyzed. The contact factor activities in plasma were measured with the corresponding specific peptide substrates.

**Results:**

The activities of contact factors (FXIIa, FXIa, plasma kallikrein) and FXa clearly increased and statistically correlated as AD progresses. We present here, for the first time, the FXIIa cut-off scores to as: > 26.3 U/ml for prodromal AD [area under the curve (AUC) = 0.783, *p* < 0.001] and > 27.2 U/ml for AD dementia (AUC = 0.906, *p* < 0.001). We also describe the cut-off scores from the ratios of CSF Aβ_1–42_ versus the contact factors. Of these, the representative ratio cut-off scores of Aβ_1–42_/FXIIa were to be: < 33.8 for prodromal AD (AUC = 0.965, *p* < 0.001) and < 27.44 for AD dementia (AUC = 1.0, *p* < 0.001).

**Conclusion:**

The activation of plasma contact system is closely associated with clinical stage of AD, and FXIIa activity as well as the cut-off scores of CSF Aβ_1–42_/FXIIa can be used as novel accurate diagnostic AD biomarkers.

**Supplementary Information:**

The online version contains supplementary material available at 10.1186/s40364-020-00258-5.

## Background

Alzheimer’s disease (AD) is the most common cause of dementia, accounting for 60–80% of all dementia patients. However, the cause of AD is poorly understood and there are no treatments currently available. To date, the only way to take care of AD is to find out in advance the onset and prevent the progression [1, 2]. Over the past decades, there have been many efforts to develop blood biomarkers that can easily and reliably detect the onset of Alzheimer’s, but it has been delayed due to the lack of reproducibility and other problems related to clinical use [3, 4]. Nevertheless, the identification of novel blood biomarkers for predicting and monitoring disease progression that can reliably detect the onset of AD at the early stage is crucial.

AD is a neurodegenerative disease characterized by amyloid-beta (Aβ) deposits in brain [5–7], and most of AD patients suffer from vascular abnormalities [8, 9] and neuroinflammation [10]. Both vascular abnormalities and inflammation can trigger neuronal death; however, the effect of Aβ on these pathologies has not been elucidated. However, a few recent reports have suggested that FXII–initiated contact system can trigger both vascular pathology and inflammation [11], and depletion of coagulation FXII can ameliorate brain pathology and cognitive impairment in AD mice [12].

The plasma contact system, which is composed of the intrinsic pathway of coagulation and the kallikrein-kinin system, plays an essential role in innate immunity [13–15]. Activation of the plasma contact system triggers several cascade systems that involve three serine protease zymogens [factor XII (FXII), factor XI (FXI), and pre-kallikrein (PKK)] and a non-enzymatic cofactor protein [high-molecular weight kininogen (HK)] [13]. The enzymatically active FXIIa activates PKK to plasma kallikrein (PK; KLKB1) that cleaves HK to release the vasoactive and proinflammatory nanopeptide, bradykinin (BK).

Several recent studies have shown a possible relationship between plasma contact system and AD. However, they did not provide any solid evidence, because of their uses of data from a very few human CSF samples or murine models [11, 12, 15–19]. Accordingly, there are still a variety of issues related to contact system and AD, including 1) Is there a close correlation between contact system and AD progression in fact?; and 2) Is it possible to obtain various cut-off scores and certain ratios for distinguishing accurately prodromal AD and AD dementia from normal subjects?

In this study, we addressed these issues and present, for the first time, the direct relationship between the contact system activation and AD progress, and the cut-off scores of FXIIa and CSF Aβ_1–42_/FXIIa ratio, which are capable of discriminating accurately prodromal AD and AD dementia from normal subjects.

## Methods

### Study participants

This study was conducted on participants in Gwangju and Jeollanam-do, Republic of Korea, from August 2015 to October 2017. All participants in this study provided their written consents, and the study protocol was approved by Chosun University Hospital Institutional Review Board (IRB file numbers 2013–12–018-068 and 2016–10–005-009). A total of 101 subjects included in the study were classified into three groups (50 normal elderly people, 23 patients with prodromal AD, and 28 patients with AD dementia) according to the clinical criteria proposed by the IWG-2 guidelines with amyloid PET [16]. However, PET positive group in normal subjects (preclinical stage of AD) and amyloid PET negative group in mild cognitive impairment (MCI) were excluded. Patients with AD dementia met the clinical criteria for Alzheimer’s disease dementia potential proposed by the NIA-AA or IWG-2 working group, and the diagnosis of MCI was also made according to the MCI criteria suggested by the NIA-AA or IWG-2 group [20, 21].

### Determination of the concentrations of Aβ_1–42_, t-Tau, p-Tau_181_, and bradykinin

The collection and storage of CSF used in this study was performed in the same process as follows [22]. The concentrations of Aβ_1–42_, t-Tau, and p-Tau_181_ in CSF were measured using INNOTEST ELISA kit (Fujirebio, Ghent, Belgium) and that of bradykinin (BK) was quantitated with Bradykinin ELISA kit (Enzo Life Sciences, Farmingdale, NY, USA) according to the protocols provided by the manufacturers.

### Measurement of the activities of FXIIa, FXIa, FXa, and PK in plasma

Plasma used in this study was collected and stored from participants according to the Molecular Medicine Ireland (MMI) guidelines for standardized biobanking [23]. To measure and make the standard curves for FXIIa, FXIa, FXa, and PK activities, various concentrations of FXIIa (0, 0.5, 1, 1.5, 2, 2.5, and 4 U/ml), FXIa (0, 0.01, 0.02, 0.05, 0.1, 0.2, and 0.4 U/ml), FXa (0, 0.01, 0.05, 0.1, 0.2, 0.5, and 1 U/ml), and PK (0, 0.02, 0.04, 0.08, 0.1, 0.2, and 0.4 U/ml) were serially diluted in phosphate buffered saline (PBS, pH 7.5). After the dilutions, 90 μl each of samples was mixed with 10 μl of corresponding synthetic peptide substrates (S-2302 for FXIIa, S-2366 for FXIa, S-2765 for FXa, and H-D-Val-Leu-Arg-AFC for kallikrein) dissolved in PBS (pH 7.5) at a final concentration of 4 mM and incubated for 30 min at 37 °C, during which the increase in absorbance at 405 nm for chromogenic substrates or in fluorescence for fluorogenic substrate (excitation 400 nm/emission 505 nm) was recorded with a 96-well plate reader (Molecular Devices, San Jose, CA, USA) as described previously [13]. All experiments were performed in triplicate. Using the data obtained, standard curves for the enzymes were then made using a sigmoidal 4 parameter curve fitting (Supplementary Fig. 1). To validate the measurement method, total 6 plasma samples and 3 different concentrations of contact factors (FXIIa, FXIa, and PK) and FXa were tested over different days.

### Statistical analysis

Statistical analysis was performed with SPSS version 24.0 (IBM Corp., Armonk, NY, USA), and analysis of variance (ANOVA) was used to compare the three groups (normal, prodromal AD, and AD dementia). Pearson’s correlation analysis was used to analyze the association between the activity of plasma contact system and the typical CSF AD biomarkers. Receiver operator characteristic (ROC) curves were generated to calculate areas under the curves (AUCs) to determine the diagnostic abilities of the contact factors and the typical CSF AD biomarkers for prodromal AD and AD dementia. The standard deviations from the mean value of the quantitative analysis or activity of each protein in the cohort used in this study was calculated as the *z*-score.

## Results and discussion

### Concentration of CSF biomarkers in AD

**Fig. 1:**
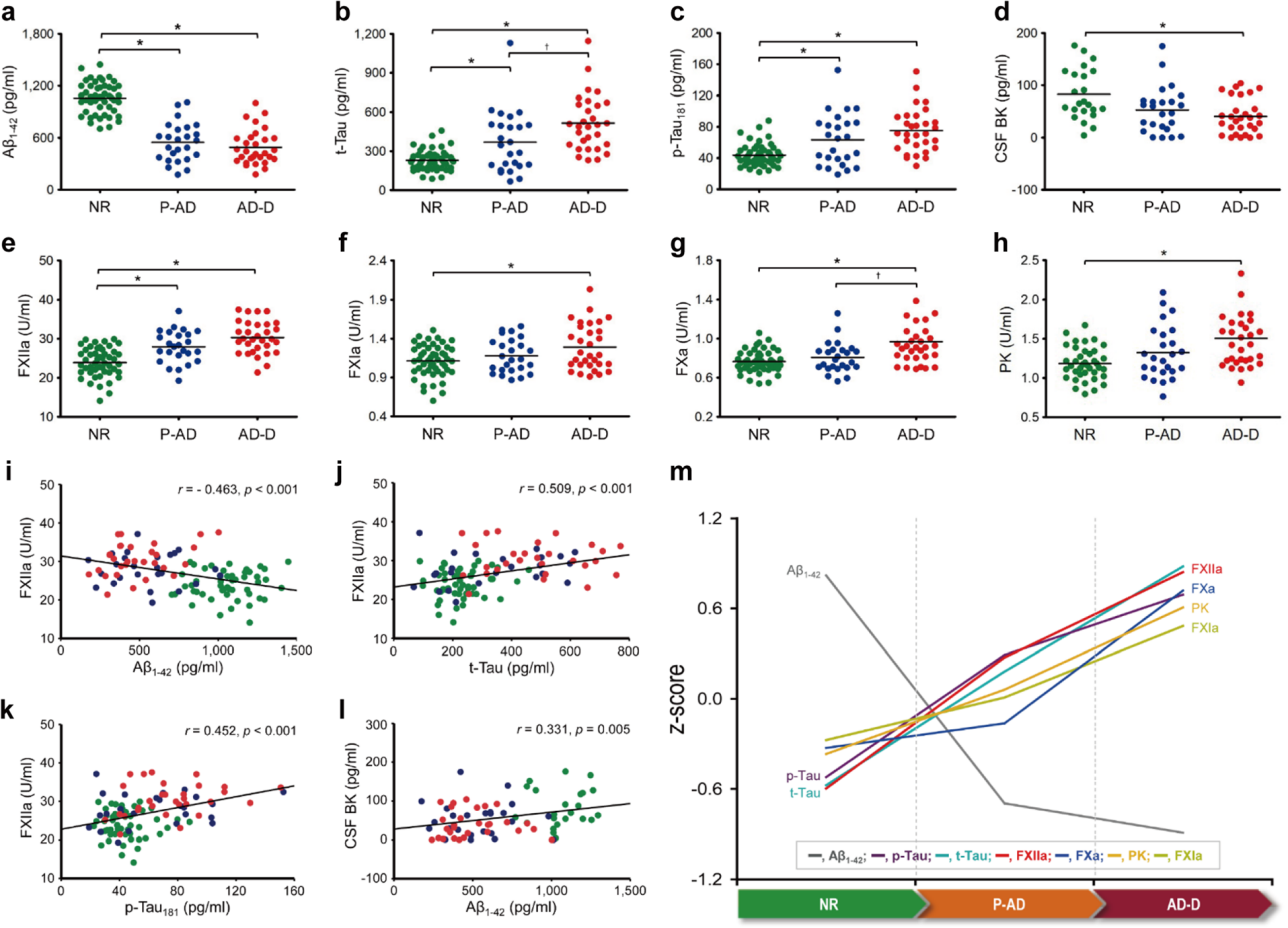
Activation of plasma contact factors with clinical stage of AD and correlation strengths between biomarkers. Concentrations of typical AD biomarkers, including Aβ_1–42_ (**a**), t-Tau (**b**), p-Tau_181_ (**c**) and BK (**d**) in CSF were measured from normal (NR), prodromal AD (P-AD), and AD dementia (AD-D) groups by ELISA. The activities of plasma contact factors such as FXIIa (**e**), FXIa (**f**), FXa (**g**), and kallikrein (**h**) were measured in the presence of 0.4 mM each of corresponding specific synthetic peptide substrates as described in Study design. Statistical analysis was performed using SPSS version 24.0 (IBM Corp., Armonk, NY, USA), and analysis of variance (ANOVA) was performed for comparisons between the three groups (NR, P-AD, and AD-D). Data are presented as mean values for each group. *: statistically significant difference between the indicated group and the NR; ^†^: statistically significant difference between the P-AD and the AD-D groups. Correlation plots of FXIIa versus Aβ_1–42_ (**i**), FXIIa versus t-Tau (**j**), FXIIa versus p-Tau_181_ (**k**), and CSF BK versus Aβ_1–42_ (**l**). Pearson’s correlation analysis was used to analyze the correlations between the activity of plasma contact factor and CSF AD biomarker as indicated, in which statistical significance was set at *p* < 0.05. In panels **a** to **l**, cyan, blue, and red colored circles represent NR, P-AD, and AD-D groups, respectively. Dynamics of biomarkers analyzed in AD pathological cascade (**m**). Lines show *z*-scores in mean values of normalized biomarker levels for each AD group

The clinical characteristics of total 101 subjects (50 normal subjects, 23 patients with prodromal AD, and 28 patients with AD dementia) and the concentrations of typical AD biomarkers, including Aβ_1–42_, t-Tau, and p-Tau_181_ in CSF and contact factors (FXIIa, FXIa, PK, and BK) and FXa in plasma samples are summarized in Table 1. As reported previously [2, 5, 24, 25], Aβ_1–42_ level in CSF decreased, while t-Tau and p-Tau181 concentrations increased with AD progression (Figs. 1a, c). The concentrations of CSF Aβ_1–42_ were estimated to be 1059, 550 and 484 pg/ml in normal, prodromal AD, and AD dementia groups, respectively (Table 1). Contrary to these, the levels of t-Tau and p-Tau_181_ increased as AD progressed, in which the concentrations of t-Tau were found to be 230, 389, and 536 pg/ml, and those of p-Tau_181_ were 44, 66, and 78 pg/ml in normal, prodromal AD, and AD dementia groups, respectively (Table 1). These results were well in accordance with reported previous studies [2, 24, 25].

**Table 1:** Demographic and biochemical characteristics of normal subjects, prodromal AD, and AD dementia

Demographic data/molecules in CSF or plasma	Total numbers of subjects	Normal	Prodromal AD	AD dementia	***p*** value
Number of subjects	101	50	23	28	
Age	101	72.0 (6.1)	71.1 (8.6)	69.4 (6.0)	0.262
Years of education	101	9.5 (5.0)	8.0 (4.7)	6.70 (3.8) ^a^	0.033
Gender, female, n (%)	101	26.0 (52.0)	12 (52.2)	17.0 (60.7)	0.736
K-MMSE score	101	26.8 (2.3)	25.3 (3.9)	17.9 (5.4) ^a, b^	< 0.001
CDR	101	0.3 (0.2)	0.5 (0.0) ^a^	0.9 (0.4) ^a, b^	< 0.001
CDR sum of boxes	101	0.5 (0.6)	1.3 (0.6)	5.1 (2.6) ^a, b^	< 0.001
GDS	101	1.6 (0.5)	3.0 (0.2) ^a^	4.1 (0.9) ^a, b^	< 0.001
B-ADL	101	20.0 (0.0)	20.0 (0.2)	19.0 (1.8) ^a, b^	< 0.001
I-ADL	101	0.04 (0.08)	0.22 (0.14) ^a^	0.63 (0.20) ^a, b^	< 0.001
**CSF biomarkers**					
Aβ_1–42_, pg/ml	101	1,059 (177)	550 (223) ^a^	484 (192) ^a^	< 0.001
t-Tau, pg/ml	101	230 (77)	389 (236) ^a^	536 (209) ^a, b^	< 0.001
p-Tau_181_, pg/ml	101	44 (14)	66 (33) ^a^	78 (28) ^a^	< 0.001
BK, pg/ml	72	83 (49)	55 (45)	39 (33) ^a^	0.002
**Plasma factors**					
FXIIa, U/ml	101	23.90 (3.70)	28.0 (3.4) ^a^	30.6 (3.7) ^a^	< 0.001
FXIa, U/ml	101	1.11 (0.21)	1.18 (0.21)	1.30 (0.30) ^a^	0.005
FXa, U/ml	101	0.77 (0.11)	0.81 (0.12)	0.97 (0.29) ^a, b^	< 0.001
Kallikrein, U/ml	101	1.20 (0.25)	1.35 (0.34)	1.54 (0.39) ^a^	< 0.001
BK, pg/ml	90	13,365 (9,305)	13,497 (7,945)	17,679 (21,186)	0.406

Data are presented as mean (± standard deviation) or number (%). *Abbreviations*: *K-MMSE* Korean Mini-Mental State Examination, *CDR* Clinical Dementia Rating, *GDS* Global Deterioration Scale, *B-ADL* Barthel Activities of Daily Living, *I-ADL* Instrumental Activities of Daily Living, *CSF* cerebrospinal fluid, *Aβ* amyloid beta-protein, *t-Tau* total Tau protein, *p-Tau* phosphorylated Tau protein, *AD* Alzheimer’s disease, *BK* bradykinin. ^a^statistically significant difference between the indicated group and the normal group; ^b^statically significant difference between prodromal AD and AD dementia groups

### Activation of contact system in AD

To measure the activation of the contact system in plasma, the assay validation standard curves were created using the recombinant contact factor (FXIIa, FXIa, and PK) and FXa enzymes. As a result, the R^2^ values of standard curves were higher than 0.95 (Supplementary Fig. 1). In addition, the coefficients of variation (CV%) were analyzed to confirm the precision and reproducibility of the measurement method for contact factor activity. As a result, the inter-assay and intra-assay CVs (%) ranged for FXIIa from 3.2 to 4.5% and from 7.2 to 12.7%, which indicated the excellent reproducibility of this method (Table 2).

**Table 2:** Overview of precision and reproducibility

Biomarker	Sample	U/ml	Intra-assay variation (***n*** = 6)			Inter-assay variation (***n*** = 4)		
			Mean	SD	CV (%)	Mean	SD	CV (%)
FXIIa	Sample 1	0.5	0.441	0.020	4.5	0.482	0.061	12.7
	Sample 2	1.5	1.458	0.047	3.2	1.521	0.165	10.9
	Sample 3	2.5	2.469	0.091	3.7	2.668	0.193	7.2
FXIa	Sample 1	0.05	0.058	0.002	2.6	0.053	0.006	11.7
	Sample 2	0.1	0.095	0.004	4.2	0.100	0.010	10.1
	Sample 3	0.2	0.199	0.008	4.1	0.197	0.019	9.4
FXa	Sample 1	0.1	0.103	0.010	9.4	0.103	0.010	10.2
	Sample 2	0.2	0.200	0.011	5.5	0.196	0.017	8.9
	Sample 3	0.5	0.510	0.023	4.5	0.515	0.059	11.5
Plasma kallikrein	Sample 1	0.1	0.125	0.004	3.0	0.110	0.012	11.3
	Sample 2	0.2	0.191	0.013	6.9	0.190	0.017	8.8
	Sample 3	0.4	0.418	0.037	8.8	0.421	0.049	11.8

Both intra (*n*=6 replicates) and inter-assay (*n*=4 assays on different day) precision were determined by comparing the mean of triplicates of three deferent concentrations of samples. The % CV is the standard deviation divided by the mean and multiplied by 100. *Abbreviations*: *SD* standard deviation, *CV* coefficients of variation, *FXIIa* active coagulation factor XII, *FXIa* active coagulation factor XI, *FXa* active coagulation factor X

The enzymatic activities of the plasma contact factors (FXIIa, FXIa, and PK) and FXa clearly increased with AD progression. The enzymatic activities for the normal, prodromal AD, and AD dementia groups were as follows: 23.9, 28.0, and 30.6 U/ml for FXIIa; 1.11, 1.18, and 1.30 U/ml for FXIa; 0.77, 0.81, and 0.97 U/ml for FXa; 1.20, 1.35, and 1.54 U/ml for PK, respectively (Table 1). Taken as a whole, the activities of contact factors (FXIIa, FXIa, or PK) and FXa had a statically difference between the three groups (normal, prodromal AD, and AD dementia groups) (Fig. 1e, h). As for BK, which is a final product of the kallikrein/kinin system [26], its concentration increased in plasma as expected, but rather decreased in CSF with the progression of AD (Table 1; Fig. 1d). The concentrations of BK in plasma were 13,365, 13,497, and 17,679 pg/ml and those in CSF were 83, 55, and 39 pg/ml in normal, prodromal AD, and AD dementia groups, respectively. These results are consistent with the those of recent reports [27] and also seemed to be related, in part, to the fact that BK can evoke blood–brain barrier (BBB) leakage and neuro-inflammation, resulting in CSF BK efflux [15, 28]. All these results suggest that the activation of plasma contact system and AD progression are obviously related, and the activities of contact factors can be used for discriminating prodromal AD and AD from normal groups.

### Analysis of correlation between AD biomarker in CSF and contact factor activity in plasma

To examine further the strength of a link between the typical CSF AD biomarkers and the contact factors, we performed correlation analyses (Fig. 1i -  l; Supplementary Table 1). As for the typical CSF AD biomarkers, the Pearson’s correlation coefficients of Aβ_1–42_ versus t-Tau and p-Tau_181_ were to be - 0.445 and - 0.359, respectively, at their *r*-values, indicating that these biomarkers are moderately correlated, under the guidelines of correlation strength [29]. In cases of contact factors, the *r*-values of FXIIa versus FXIa, FXa, and PK were to be 0.8, 0.692, and 0.59, respectively, suggesting that these factors are correlated statistically in significant level (Supplementary Table 1). Prominently, FXIIa was negatively correlated to CSF Aβ_1–42_ (*r* = − 0.463) and positively to both t-Tau (*r* = 0.509) and p-Tau_181_ (*r* = 0.452) in all moderate relationships (Fig. 1i - l; Supplementary Table 1). All these results suggest that the activation of contact system is certainly correlated to AD progression as reported previously [17].

The changes in the *z*-values of typical CSF AD biomarkers and plasma contact factors as AD progresses were also analyzed (Fig. 1m). As expected [5, 12, 16, 30], the *z*-score of Aβ_1–42_ decreased, whereas those of t-Tau and p-Tau_181_ increased in the order of normal, prodromal AD, and AD dementia groups (Fig. 1m). As for plasma contact factors, the scores of FXIIa, FXIa, FXa, and PK noticeably increased (Fig. 1m), whereas CSF BK decreased (data not shown) in the AD pathological cascade. These results strongly suggest that plasma contact system is closely associated with AD progression, and its degree of activation can reflect the disease progression.

### Analysis of potential as a plasma biomarker for AD diagnosis

**Fig. 2:**
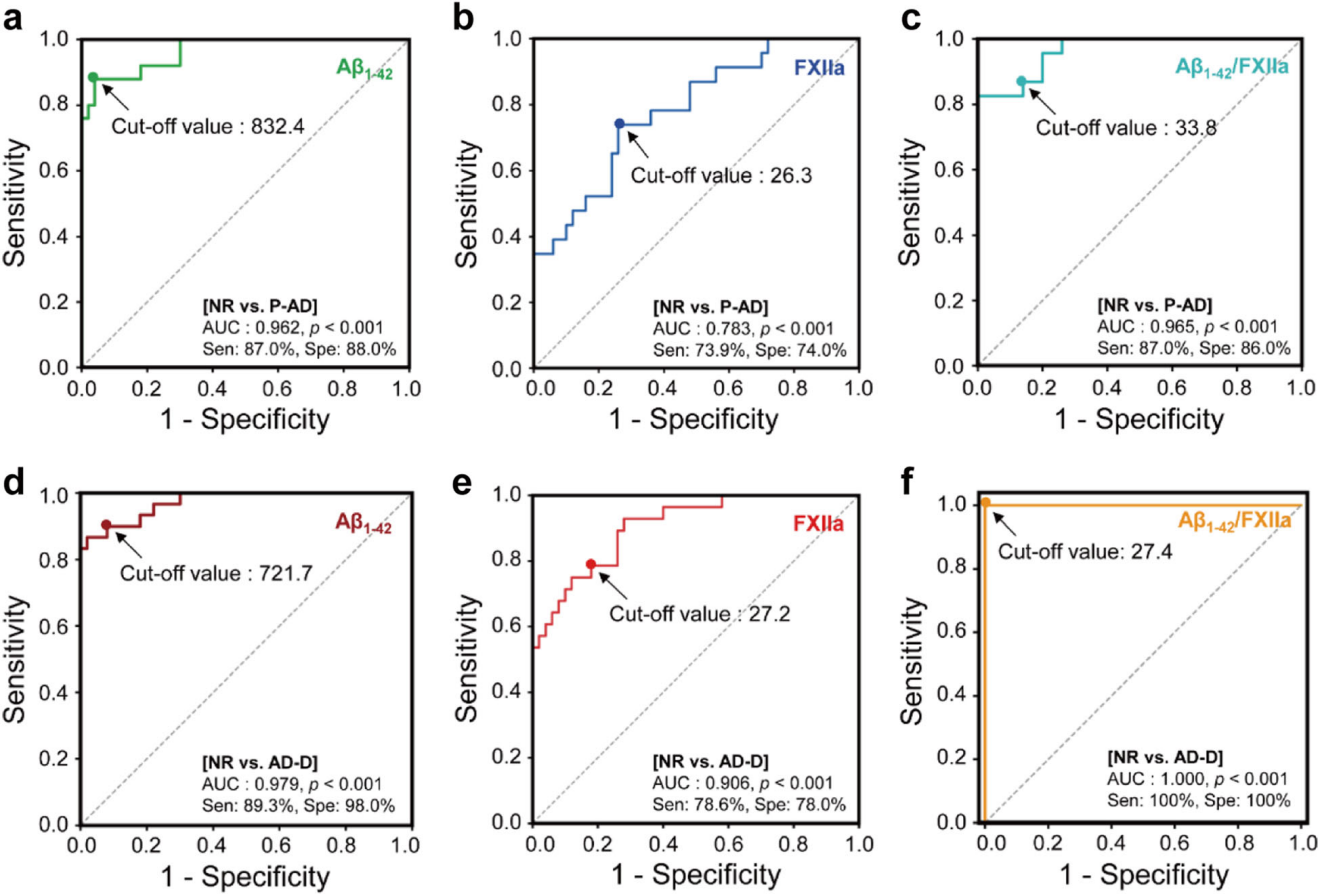
Representative cut-off values of contact factors and CSF Aβ_1–42_/contact factor ratios. Cut-off values of Aβ_1–42_ (**a**), FXIIa (**b**), and CSF Aβ_1–42_/FXIIa ratio (**c**) capable of discriminating prodromal AD from normal. Cut-off values of Aβ_1–42_ (**d**), FXIIa (**e**), and CSF Aβ_1–42_/FXIIa ratio (**f**). ROC curves are shown for each marker, in which arrows indicate cut-off values capable of distinguishing P-AD and AD-D groups from NR

We generated receiver operating characteristic (ROC) curves to analyze the potential of each protein for use as a new biomarker for AD diagnosis (Fig. 2 and Table 3). The ROC curves and the areas under the curves (AUCs) [28, 31] showed that Aβ_1–42_ can discriminable in highly accurate for both prodromal AD (AUC = 0.962; cut-off value = < 832.4 pg/ml) and AD dementia (AUC = 0.979; cut-off value = < 721.7 pg/ml) from normal (Fig. 2a, d; Table 3). Among the contact factors, only FXIIa seemed to have a capability able to discriminate both prodromal AD (AUC = 0.783; cut-off value = > 26.3 U/ml) and AD dementia (AUC = 0.906; cut-off value = > 27.2 U/ml) from normal (Fig. 2b,e; Table 3). These results indicate that the contact factor FXIIa can be a new plasma biomarker for diagnosing prodromal AD in acceptable and AD dementia in very accurate from normal.

**Table 3:** Cut-off scores and sensitivity/specificity values of fluid biomarkers for discriminating the prodromal AD or AD dementia from normal subjects

Characteristics	Normal (***n*** = 50) vs. Prodromal AD (***n*** = 23) groups					Normal (***n*** = 50) vs. AD dementia (***n*** = 28) groups				
	Cut-off	Sen (%)	Spe (%)	AUC	***p*** value	Cut-off	Sen (%)	Spe (%)	AUC	***p*** value
**Plasma factors**										
FXIIa activity, U/ml	> 26.3	73.9	74.0	0.783	< 0.001	> 27.2	78.6	78.0	0.906	< 0.001
FXIa activity, U/ml	> 1.13	52.2	52.0	0.566	0.367	> 1.17	57.1	58.0	0.659	0.020
FXa activity, U/ml	> 0.76	56.5	54.0	0.573	0.316	> 0.82	71.4	70.0	0.779	< 0.001
Kallikrein activity, U/ml	> 1.21	56.5	56.0	0.609	0.138	> 1.29	64.3	64.0	0.772	< 0.001
**CSF biomarkers**										
Aβ_1–42_ levels, pg/ml	< 832.4	87.0	88.0	0.962	< 0.001	< 721.7	89.3	98.0	0.979	< 0.001
t-Tau levels, pg/ml	> 252.6	65.2	66.0	0.701	0.006	> 315.3	85.7	86.0	0.954	< 0.001
p-Tau_181_ levels, pg/ml	> 46.1	65.2	64.0	0.690	0.009	> 46.1	78.6	78.0	0.870	< 0.001
BK levels, pg/ml	> 46.1	54.5	56.5	0.656	0.073	> 52.8	74.1	73.9	0.783	0.001
**Ratios**										
Aβ_1–42_ / FXIIa	< 33.8	87.0	86.0	0.965	< 0.001	< 27.4	100.0	100.0	1.000	< 0.001
Aβ_1–42_ / FXIa	< 742.5	82.6	82.0	0.930	< 0.001	< 635.4	96.4	96.0	0.993	< 0.001
Aβ_1–42_ / FXa	< 1,091	82.6	82.0	0.933	< 0.001	< 941.8	96.4	96.0	0.994	< 0.001
Aβ_1–42_ / Kallikrein	< 680.9	87.0	86.0	0.943	< 0.001	< 584.8	96.4	96.0	0.994	< 0.001

Statistically-derived optimal cut-off value was determined with the best balance between sensitivity (Sen) and specificity (Spe) values. Discrimination of prodromal AD and AD dementia from normal group was evaluated by receiver operator characteristics (ROC) curve analysis and quantified by the area under the curve (AUC) using SPSS software version 24.0

In particular, the CSF Aβ_1–42_/FXIIa ratio showed very accurate for prodromal AD (AUC = 0.965; cut-off value = < 33.8) and perfect diagnostic abilities for AD dementia (AUC = 1.0; cut-off value = < 27.44) (Fig. 2c, f; Table 3). Taken together, these results suggest that, 1) FXIIa can be used as a plasma biomarker for early diagnosis of prodromal AD, and 2) use of the Aβ_1–42_/FXIIa ratio improves diagnostic accuracy.

## Conclusions

Based on the results, we conclude that 1) activation of plasma contact system is not only correlated with AD progression, but also is available for AD diagnosis; 2) the degree of AD progress can be quickly determined by measuring the activities of contact factors in blood plasma; 3) among the contact factors, FXIIa can be used as a new plasma biomarker for diagnosing prodromal AD and AD dementia at early stage, and 4) the use of Aβ_1–42_/FXIIa ratio improves diagnostic accuracy for discriminating prodromal AD and AD dementia from normal.

## Supplementary Information

**Additional file 1: Supplementary Figure 1.** The standard curves for contact factors activities (FXIIa, FXIa, Fxa, and plasma kallikrein). The curves were fitted by sigmoidal 4 parameter curve of data points.

**Additional file 2:: Supplementary Table 1.** Correlation of CSF AD biomarkers and plasma contact factors.

## Data Availability

For original data, please contact jsplee@chosun.ac.kr. Detailed data on correlation and cut-off scores may be found in “Supplemental Table 1” available with the online version of this article.

## Abbreviations

AD: Alzheimer’s disease
FXIIa: Factor XIIa
Aβ,: Amyloid-beta
FXI: Factor XI
PKK: Prekallikrein
HK: High-molecular weight kininogen
PK: Plasma kallikrein
BK: Bradykinin
CSF: Cerebrospinal fluid
BBB: Blood-brain barrier
