# Supplementary figures and images for: Plasma contact factors as novel biomarkers for diagnosing Alzheimer’s disease
Source: Biomark Res. 2021 Jan 9;9:5. doi: 10.1186/s40364-020-00258-5 (PMC7796542; doi:10.1186/s40364-020-00258-5)

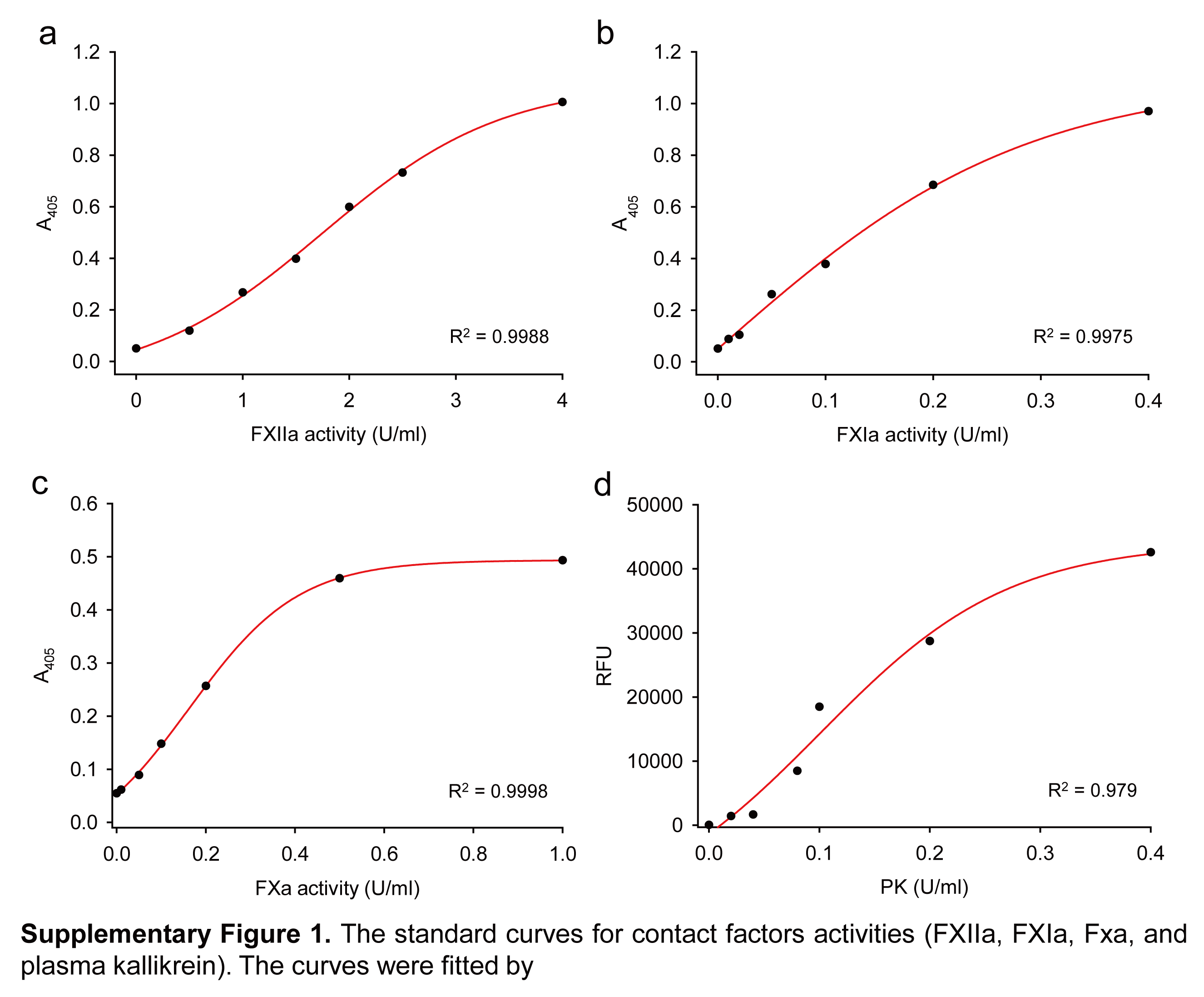

Supplement: Supplementary file 1 — Additional file 1: Supplementary Figure 1. The standard curves for contact factors activities (FXIIa, FXIa, Fxa, and plasma kallikrein). The curves were fitted by sigmoidal 4 parameter curve of data points. [file 40364_2020_258_MOESM1_ESM.tif]
