# Supplementary material for: Plasma contact factors as novel biomarkers for diagnosing Alzheimer’s disease
Source: Biomark Res. 2021 Jan 9;9:5. doi: 10.1186/s40364-020-00258-5 (PMC7796542; doi:10.1186/s40364-020-00258-5)
Supplement: Supplementary file 2 — Additional file 2:: Supplementary Table 1. Correlation of CSF AD biomarkers and plasma contact factors. [file 40364_2020_258_MOESM2_ESM.docx]

**Supplementary Table 1. Correlation of CSF AD biomarkers and plasma contact factors**

| **Molecules** | **CSF AD biomarker** | | | **Plasma contact factors** | | | | | | | |
| --- | --- | --- | --- | --- | --- | --- | --- | --- | --- | --- | --- |
|  | **Aβ_1-42_** | **t-Tau** | **p-Tau_181_** |  | **FXIIa** | **FXIa** | **FXa** | **Kallikrein** | **CSF BK** | **Plasma BK** |  |
| Aβ_1-42_ | 1 | **- 0.445^†^** | **- 0.359^†^** |  | **- 0.463^†^** | - 0.181 | - 0.305^†^ | - 0.294^†^ | **0.331^†^** | - 0.026 |  |
| t-Tau |  | 1 | **0.910^†^** |  | **0.509^†^** | 0.258^†^ | 0.257^†^ | 0.260^†^ | - 0.002 | 0.164 |  |
| p-Tau_181_ |  |  | 1 |  | **0.452^†^** | 0.288^†^ | 0.222^*^ | 0.288^†^ | - 0.002 | 0.174 |  |
| FXIIa |  |  |  |  | 1 | **0.800^†^** | **0.692^†^** | **0.590^†^** | - 0.226^*^ | 0.249^*^ |  |
| FXIa |  |  |  |  |  | 1 | **0.636^†^** | **0.523^†^** | - 0.114 | 0.167 |  |
| FXa |  |  |  |  |  |  | 1 | **0.623^†^** | - 0.265^*^ | 0.205 |  |
| Kallikrein |  |  |  |  |  |  |  | 1 | - 0.126 | 0.228^*^ |  |
| CSF BK |  |  |  |  |  |  |  |  | 1 | - 0.033 |  |
| Plasma BK |  |  |  |  |  |  |  |  |  | 1 |  |

Data are presented as Pearson’s correlation coefficient (*r*). Bold values indicate statistically significant correlations (*, *p* < 0.05; ^†^, *p* < 0.01). Abbreviations: Aβ, amyloid-beta protein; t-Tau, total Tau; protein; p-Tau, phosphorylated Tau protein; AD, Alzheimer’s disease; FXIIa, active coagulation factor XII; FXIa, active coagulation factor XI; FXa, active coagulation factor X; BK, bradykinin.
